# Calcified intracranial epidermoid cyst presented during pregnancy: A case report

**DOI:** 10.1016/j.radcr.2025.04.103

**Published:** 2025-05-15

**Authors:** Parisa Pishdad, Amirhossein Soltani, Amirmehdi Ghanbarzadeh, Shakiba Houshi, Mohsen Salimi

**Affiliations:** a Department of Radiology, Shiraz University of Medical Sciences, Shiraz, Iran; b Medical Imaging Research Center, Shiraz University of Medical Sciences, Shiraz, Iran; c School of Medicine, Shiraz University of Medical Sciences, Shiraz, Iran; d Isfahan Neurosciences Research Center, Isfahan University of Medical Sciences, Isfahan, Iran

**Keywords:** Brain tumor, Calcified epidermoid cyst, Intracranial epidermoid cyst, Pregnancy

## Abstract

We present the case of a 29-year-old female with a history of an intracranial tumor, initially diagnosed at age 22, who presented during her first pregnancy at 23 weeks with a seizure episode. MRI revealed a nonenhancing mass in the middle cranial fossa with high T1-weighted signal intensity. Despite concerns regarding the potential for eclampsia, the patient was conservatively managed with seizure prophylaxis, and a cesarean section was performed at 38 weeks’ gestation. Over the following years, the patient experienced stable imaging findings and mild headaches but later developed worsening symptoms due to mass effect, requiring a Ventriculoperitoneal shunt and subsequent tumor resection. Pathology confirmed the diagnosis of an epidermoid cyst. This case highlights the importance of a multidisciplinary approach to managing intracranial lesions in pregnancy, as early diagnosis and careful coordination between obstetrics, neurology, and neurosurgery teams are crucial to ensuring optimal maternal and fetal outcomes. Additionally, it underscores the need for long-term follow-up in patients with brain lesions during pregnancy, as complications may arise years later, necessitating timely interventions. Notably, this case also demonstrates unique characteristics of an epidermoid cyst with high T1-weighted signal and hyperdensity on CT that remained unchanged over time, favoring the diagnosis of a calcified epidermoid cyst—a rare entity.

## Introduction

Intracranial epidermoid cysts (IEC) are rare, benign, slow-growing extra-axial tumors, making up about 0.3% to 1.8% of all primary intracranial tumors [1]. Although these tumors grow slowly, they often attach to vital neurovascular structures. They can cause compression due to the mass effect, leading to symptoms like seizures, headaches, and diplopia, along with substantial morbidity and neurological dysfunction [2]. Magnetic resonance imaging (MRI) is the primary diagnostic method, with diffusion-weighted imaging playing a key role in differentiating IECs from other lesions. On MRI, IECs typically display signal characteristics resembling cerebrospinal fluid on both T1- and T2-weighted sequences and usually do not have contrast enhancement [3,4]. Intracranial tumors during pregnancy are a rare occurrence that requires a specialized multidisciplinary approach to optimize outcomes for the mother and the developing fetus [5,6]. We present a case of a calcified IEC with a unique signal intensity pattern that first manifested during pregnancy as a seizure episode, along with a detailed overview of its imaging, diagnosis, and management strategies.

## Case presentation

A 29-year-old female with a history of brain tumor (first diagnosed at age 22) presented to our center with progressively worsening severe headaches. Her symptoms began at age 22, during the 23rd week of her first pregnancy, when she experienced an episode of loss of consciousness lasting only a few seconds, which led to a fall. She was fully awake afterward with no neurological deficits. She was brought to the Emergency Department, where her level of consciousness was normal (Glasgow Coma Scale (GCS): 15). A neurological examination was unremarkable and vital signs were stable. The patient was admitted for further evaluation, especially fetal assessment. Routine laboratory tests and ECG were normal, and fetal sonography showed no abnormalities with normal fetal activity. A nonstress test (NST) was also performed and was normal.

A few hours later, just before discharge, the patient had a tonic-clonic seizure lasting less than 1 minute that stopped on its own. At the time, the patient had no significant past medical or surgical history. She was not on any medications and had no history of smoking or alcohol use. Her family history was also unremarkable.

Although her blood pressure was within normal range (120/80 mmHg), with a strong possibility of eclampsia as a differential diagnosis, the patient was admitted to the high-risk pregnancy ward. Heart monitoring and pulse oximetry were started. She received a loading dose of 4g magnesium sulfate IV over 20 minutes, followed by a maintenance infusion of 1g per hour. Fetal monitoring (NST), detailed ultrasound, and biophysical profile assessment were repeated, and all results appeared normal.

To assess for possible liver involvement and other conditions, such as HELLP syndrome, routine laboratory tests were repeated. In addition, liver function tests (LFT), coagulation profile, uric acid, creatinine, and a 24-hour urine collection for creatinine and protein were ordered. All results were completely normal.

For further evaluation of other potential causes of seizures, a neurology consult was requested. Based on the findings and the opinions of both the obstetrics and neurology teams, the patient was scheduled for a brain MRI for further assessment.

**Fig. 1:**
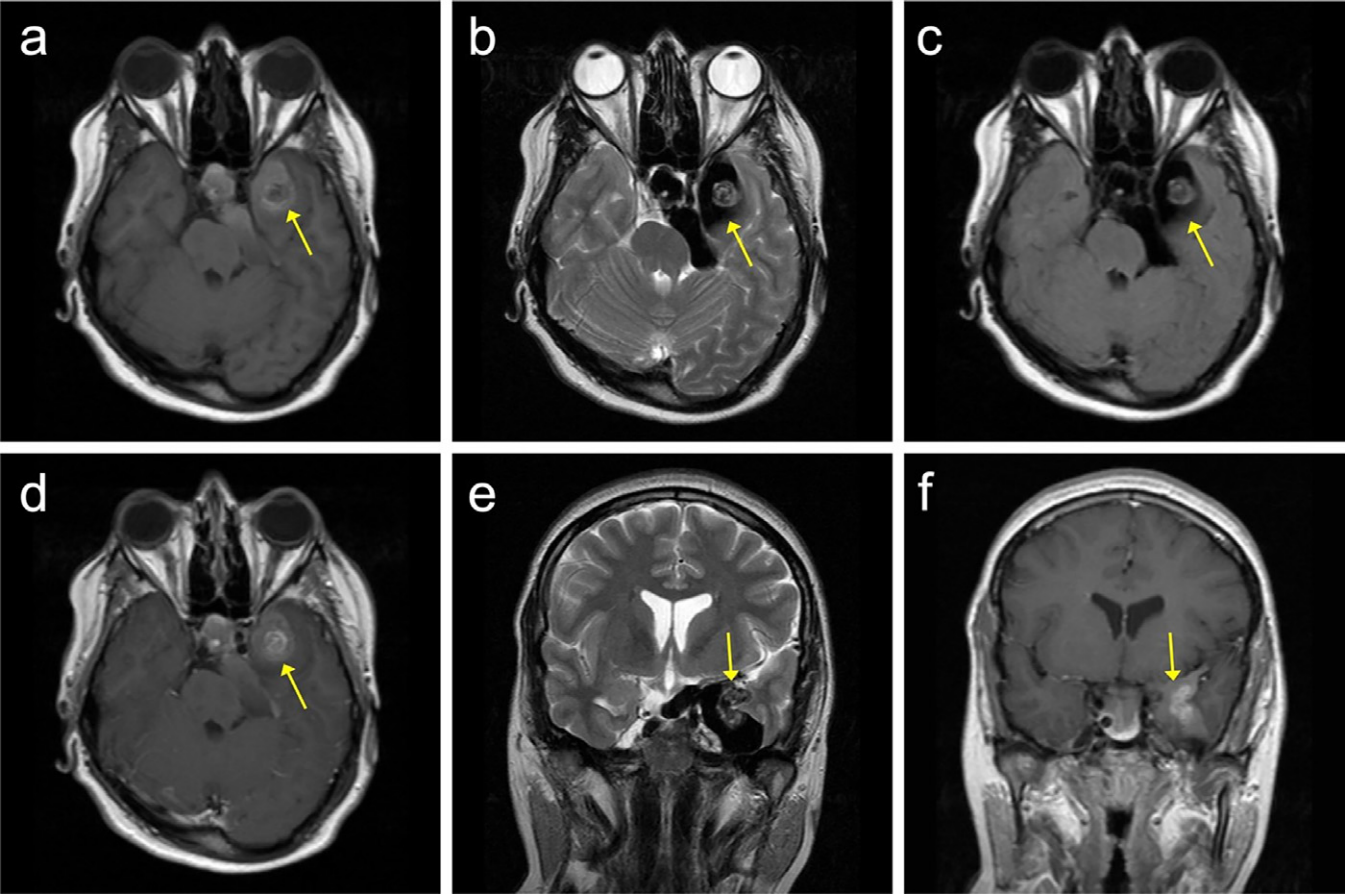
Brain MRI with and without GAD. (A) Axial T1W (B) Axial T2W (C) Axial T2 FLAIR (D) Axial T1W with GAD (E) Coronal T2W (F) Coronal T1W with GAD. Evidence of a lobulated nonenhancing heterogeneous mass with High signal intensity on T1 and low signal intensity on T2 and FLAIR images is present in the left middle cranial fossa, with pressure effects on the left temporal lobe and left side of the pons. The epidermoid cyst is indicated with a yellow arrow in all sequences.

After obtaining the patient's consent, a brain MRI was performed, which revealed a lobulated, nonenhancing mass with High signal on T1 and low signal on T2 and FLAIR images. The mass was located in the middle cranial fossa. It was causing a pressure effect on the left temporal lobe and left side of the pons, with close contact with the optic chiasm (Fig. 1). Based on these characteristic findings, the differential diagnosis of an epidermoid cyst or meningioma was considered.

After consultation with the neurosurgeon and evaluation by a multidisciplinary team (neurologist, neurosurgeon, obstetrician), no surgical intervention was proposed. The patient was discharged with levetiracetam 500 mg twice a day as seizure prophylaxis. She was provided with close monitoring and scheduled for regular outpatient visits with the obstetrics team.

All visits were normal, and the fetus was monitored for several weeks. Due to concerns regarding increased intracranial pressure during vaginal delivery and the high-risk nature of the pregnancy, the patient underwent a cesarean section at 38 weeks' gestation. The operation was performed successfully and uneventfully. Both the mother and the newborn were in good condition after the cesarean section. Levetiracetam and neurosurgery follow-ups were maintained for the patient.

**Fig. 2:**
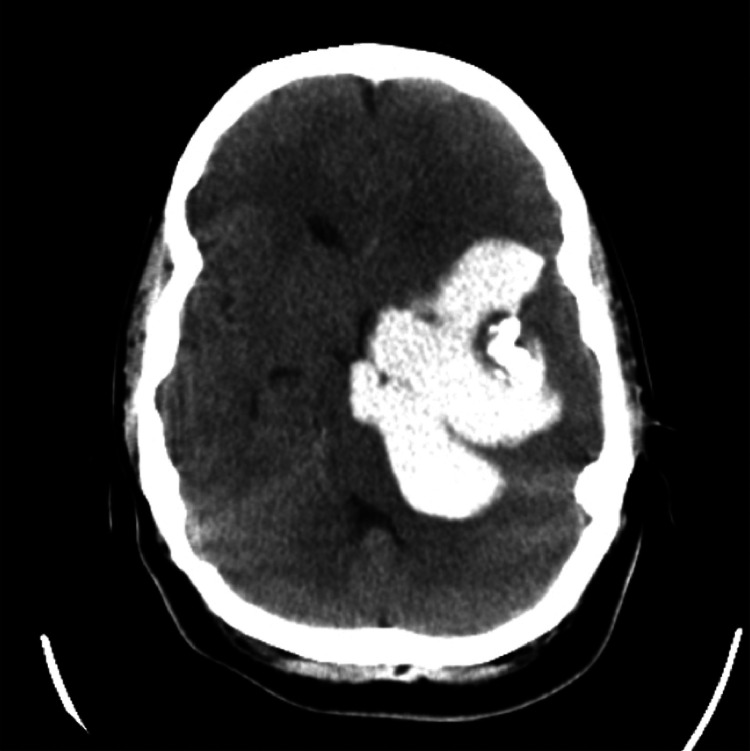
Noncontrast axial brain CT showing an extra-axial, lobulated hyperdense lesion with coarse internal calcifications.

The patient was followed for years, and her imaging remained stable with no changes (Fig. 2). There were no further episodes of seizures, and no neurological deficits or weakness developed. She only experienced mild episodes of headaches, which were managed with conservative treatment.

Seven years after the initial diagnosis, at the age of 29 (current report), the patient’s headaches worsened and began interfering with her daily activities. Except for mild hydrocephalus, MRI showed no significant changes. Subsequently, the patient developed episodes of nausea, vomiting, vertigo, and a severe, progressive headache. Based on the neurosurgeon's opinion, the patient was scheduled for insertion of a Ventriculoperitoneal (VP) shunt to resolve the mass effect and hydrocephalus. Therefore, a VP shunt was inserted into the right lateral ventricle, and the procedure was uneventful.

Over the next 6 months, the patient's headaches did not resolve and even worsened. After consulting with the neurosurgeon and considering the patient's preference, she was scheduled for craniotomy and tumor resection. A left temporal craniotomy and subtemporal gross total resection of the extra-axial mass were performed, along with a secondary dural graft and cranioplasty using 3 plates and 8 screws. The surgery went smoothly, and the patient recovered well, though she experienced amnesia with short-term memory loss. No other cognitive changes were observed. The tumor sample was sent for pathology, and the results confirmed the diagnosis of an epidermoid cyst.

After a few days of hospitalization and continuous monitoring of the patient’s cognitive status, her memory problems resolved. Follow-up computed tomography (CT) scans showed no further complications. The patient was discharged from the hospital with the following medications: clopidogrel 100 mg 3 times a day (antiplatelet), rivaroxaban 10 mg once a day (anticoagulant), dexamethasone 2 mg twice a day (anti-inflammatory), and cefalexin 500 mg every 6 hours for 2 weeks (antibiotic), and levetiracetam 1000 mg twice a day (antiepileptic).

### Patient outcome

**Fig. 3:**
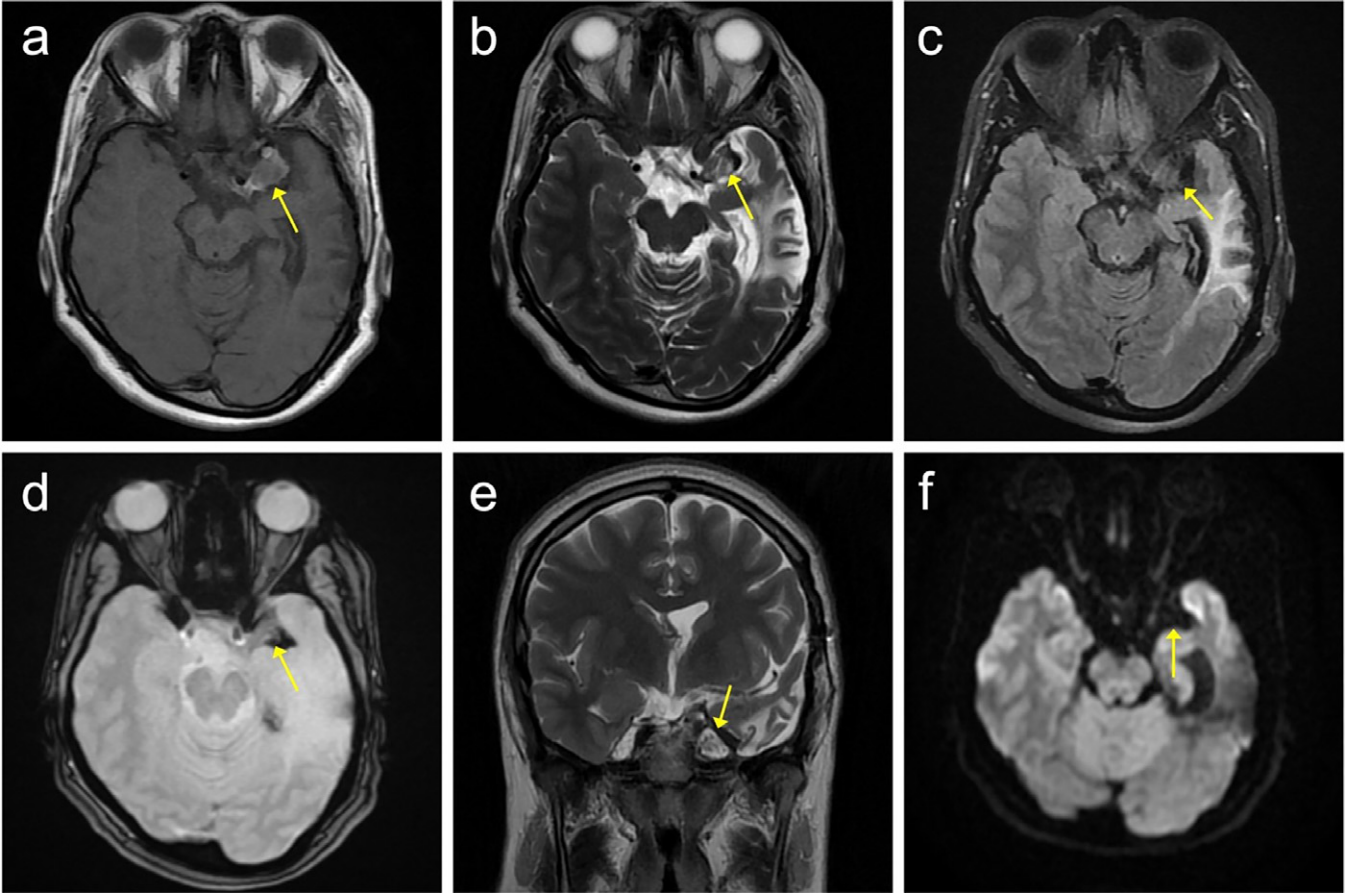
Follow-up Brain MRI performed 1 year after surgery. (A) Axial T1W (B) Axial T2W (C) Axial T2 FLAIR (D) Axial T2 GRE (E) Coronal T2W (F) DWI. A high T1 and low T2 signal intensity lesion is observed in the left middle cranial fossa, without enhancement, suggesting a possible remnant of calcification from a previous tumor. The remnant tumor is indicated by the yellow arrow in all sequences.

Since then, the patient's follow-ups have been uneventful. She occasionally reports experiencing short-term memory loss, but it is not permanent, and she has mild headaches from time to time. Aside from these, no complications have been observed. Imaging studies show no significant changes, with only a remnant of the previous calcified lesion remaining (Fig. 3).

## Discussion

We reported a case of an IEC that presented during pregnancy. Based on our broad search, there was only 1 report that mentioned this occurrence during pregnancy, but in that report, the patient was symptomatic before pregnancy [7]. The nature of pregnancy often complicates the diagnosis of intracranial tumors, as many symptoms such as headache, nausea, and vomiting may be mistakenly attributed to normal pregnancy-related changes. This can lead to a misdiagnosis, where the underlying tumor remains unidentified [8]. In our case, the patient was initially set to be discharged after a full evaluation for an episode of loss of consciousness, with no abnormalities found. However, during the process, the patient experienced a seizure, a highly dangerous occurrence during pregnancy. This emphasizes the critical importance of thoroughly evaluating neurological symptoms in pregnant patients, as they can mask potentially life-threatening conditions.

Several studies have linked pregnancy as a potential factor in the growth of intracranial tumors, though this remains controversial and is dependent on tumor type and pathophysiology [6,9,10]. Given the rarity of such co-occurrence, further studies and future research are needed to explore these links. Nevertheless, pregnancy can modulate the pathophysiology and clinical expression of these tumors due to hormonal fluctuations, alterations in immune tolerance, and hemodynamic changes that occur during gestation [11,12]. This could explain the aggressive and sudden presentation of the IEC in our case, manifesting as a seizure in a previously asymptomatic patient. In such cases, a multidisciplinary approach is crucial to ensure accurate diagnosis and optimal management [13], as demonstrated in our case.

As mentioned earlier, MRI is the modality of choice for diagnosing IECs. MRI is generally regarded as safe for the fetus during pregnancy [14]; however, the administration of gadolinium remains controversial. While some studies suggest potential harm, others consider it safe. In such cases, it is crucial to avoid gadolinium unless absolutely necessary for diagnosis [15]. Patient autonomy must be respected by ensuring a thorough discussion of potential risks and benefits, allowing the patient to make an informed decision [13].

The primary treatment for IECs is surgical resection. Gross total resection is essential for reducing the risk of recurrence, as studies have shown an association between incomplete capsule removal and tumor recurrence [16,17]. However, due to the close adherence of these tumors to critical neurological structures, achieving gross total resection is not always feasible or advisable [2]. In our case, gross total resection was performed, yet remnants of calcified tissue were still observed. In IECs, postsurgical recurrence remains a risk even after gross total resection, particularly when residual tissue is visible on postoperative MRI [18]. Therefore, close follow-up is essential to monitor for potential recurrence and ensure timely intervention if needed. Although extremely rare, postsurgical transformation of IECs into malignancy should be considered and monitored during follow-ups to ensure early detection and appropriate management [[19], [20], [21]]. The appearance of peritumoral edema or notable enhancement on MRI should raise suspicion of malignant transformation and alert the neurosurgeon to the need for further evaluation and close monitoring [19]. Due to the benign nature of IECs, cases generally have excellent survival rates, with many studies reporting zero mortality [22].

Epilepsy imaging primarily involves detecting structural abnormalities on MRI, differentiating between pathological lesions and normal variations, aligning imaging findings with clinical epilepsy history, and determining lesion boundaries for surgical preparation [23]. Differential diagnosis in this case to consider could include meningioma, glioma, and dermoid cyst. Additionally, the target-like appearance seen in our patient's imaging is similar to the imaging characteristics of some hemangioma cases [24]; however, based on pathology, the lesion was identified as an IEC.

As mentioned earlier, on MRI, intracranial epidermoid cysts (IECs) typically exhibit signal characteristics similar to cerebrospinal fluid on both T1- and T2-weighted sequences and usually do not show contrast enhancement. However, it is important to note that some cases display variations, as seen in our case, which presented a more nonspecific signal intensity pattern—hyperintense on T1 and hypointense on T2. This pattern is often associated with iron deposition from a prior hemorrhagic event or calcification at the lesion site. Notably, in our case, the extent and signal intensity of the lesion remained unchanged over time across multiple imaging studies, suggesting calcification within the lesion. Calcification in IECs is observed in a low proportion of cases (10%) [25], which further highlights the uniqueness of our described case.

In our case, diagnosing an intracranial lesion during pregnancy posed unique challenges. At 23 weeks’ gestation, the initial differential diagnosis was eclampsia due to the seizure occurrence. However, the absence of hypertension and normal laboratory findings necessitated further neurological evaluation. Given the potential benign nature of the disease and the risks associated with surgery during pregnancy, the multidisciplinary team opted for a nonsurgical approach with close observation until the end of pregnancy. This underscores the importance of individualized management, a multidisciplinary approach, and cautious monitoring in balancing maternal and fetal well-being. The choice to perform a cesarean delivery at 38 weeks was driven by concerns over possible intracranial pressure elevation during labor, which could have resulted in complications such as herniation or recurrent seizures. This underscores the need for personalized delivery planning in pregnant patients with intracranial tumors, carefully weighing the risk of maternal neurological decline against obstetric factors.

Although imaging remained stable and no seizures recurred for several years, the patient eventually experienced worsening headaches and signs of progressive vascular involvement. Encasement of major arteries, including the basilar and middle cerebral arteries, indicated chronic compression from the mass effect, increasing ischemic risk. The subsequent onset of hydrocephalus required VP shunt placement, which initially relieved intracranial pressure but failed to resolve the headaches. This progression highlights the need for long-term follow-up in patients with intracranial cystic lesions, as symptoms may evolve despite initial stability. Ultimately, surgical intervention became necessary due to persistent and worsening symptoms. The patient's postoperative short-term memory loss was likely due to temporary disruption of temporal lobe structures and perilesional edema, both of which resolved over time. Further research is needed to investigate the cognitive effects of total resection in IECs.

## Conclusion

IECs can remain asymptomatic for years but may become clinically significant in pregnancy due to physiological changes. This case underscores the importance of considering alternative causes of seizures in pregnancy beyond eclampsia and highlights the role of multidisciplinary management in optimizing maternal and fetal outcomes. Long-term monitoring is essential, as delayed complications may arise, requiring timely intervention.

## Patient consent

Written consent was obtained from the patient for the publication of this case report and accompanying images. All identifiable details have been omitted to ensure patient confidentiality.
